# Stability and rheology of dilute TiO_2_-water nanofluids

**DOI:** 10.1186/1556-276X-6-273

**Published:** 2011-03-31

**Authors:** Vera Penkavova, Jaroslav Tihon, Ondrej Wein

**Affiliations:** 1 Institute of Chemical Process Fundamentals, Academy of Sciences of the Czech Republic, Rozvojova 135, 165 02 Prague 6, Czech Republic

## Abstract

The apparent wall slip (AWS) effect, accompanying the flow of colloidal dispersions in confined geometries, can be an important factor for the applications of nanofluids in heat transfer and microfluidics. In this study, a series of dilute TiO_2 _aqueous dispersions were prepared and tested for the possible presence of the AWS effect by means of a novel viscometric technique. The nanofluids, prepared from TiO_2 _rutile or anatase nanopowders by ultrasonic dispersing in water, were stabilized by adjusting the pH to the maximum zeta potential. The resulting stable nanofluid samples were dilute, below 0.7 vol.%. All the samples manifest Newtonian behavior with the fluidities almost unaffected by the presence of the dispersed phase. No case of important slip contribution was detected: the Navier slip coefficient of approximately 2 mm Pa^-1 ^s^-1 ^would affect the apparent fluidity data in a 100-μm gap by less than 1%.

## Background

Bulk rheological properties of nanofluids (shear viscosity [1,2], yield stress [3-7], and complex modulus [8]) can be important factors for some applications (e.g., convective heat transfer [9,10], and filtration [5]) and can also provide some correlations with other properties, such as volumetric particle concentration [1,2], thermal conductivity [11,12], or *ξ*-potential [3-6].

On the other hand, there are processes with a dominant microscopic length scale, such as small Nernst diffusion thickness in heat/mass transfer [13], small hydraulic radius in microfluidics [14-17], small pore diameter in filtration [5], etc., where the bulk rheology characteristics should be completed using another kind of information. In some cases, two-scale description (particle size or inter-particle distance vs. hydraulic radius) is useful [15]. In other cases, an additional macroscopic interfacial property, like apparent wall slip (AWS) velocity [18,19], could provide the missing information.

In this study, we examine experimentally the AWS effect in dilute TiO_2_-water nanofluids, using a novel AWS viscometric technique [19].

## Experimental procedure

### Preparation and stability of the samples

Sample nanofluids were prepared by dispersing a nanopowder in an aqueous electrolyte solution (the base solution). The TiO_2 _nanopowders (A1, A2, A3, R1, and R2) used in this study are specified in Table 1. The base solutions with adjusted pH values were prepared by adding HCl or NaOH to demineralized water with a possible content of dissolved gases.

**Table 1:** Nanopowders used for the preparation of nanofluids

Powder	Mineral	Source	Density (g cm-3)	Max. size (nm)
A1	TiO_2 _anatase	Aldrich	3.90	25
A2	TiO_2 _anatase	ICPF^a^	3.90	40
A3	TiO_2 _anatase	ICPF^a^	3.90	20
R1	TiO_2 _rutile	Aldrich	4.17	100
R2	TiO_2 _rutile	Precheza^b^	4.17	100

^a^ICPF - nanopowder for photocatalytic application supplied by Department of Catalysis of ICPF ASCR, Prague

^b^Precheza - commercial pigment, produced by Prerov Chemical Works, Czech Republic

**Figure 1:**
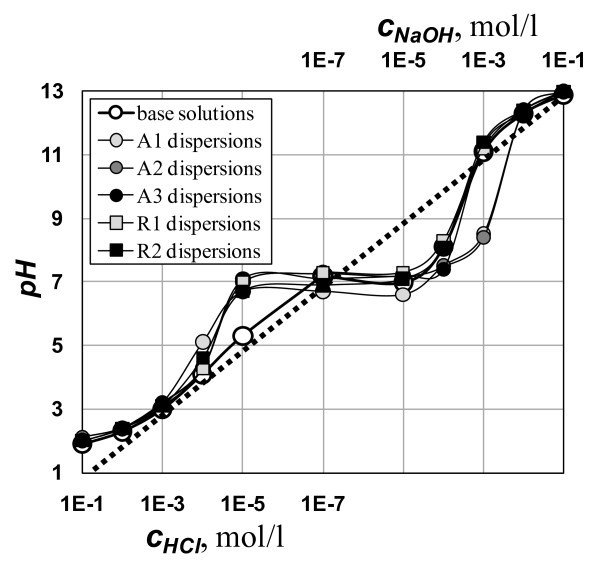
**Titration curves of the tested samples**. Dotted line shows an idealized titration curve. Deviations for the individual samples are due to dissociation of hydrated TiO_2 _and dissolved CO_2_.

**Figure 2:**
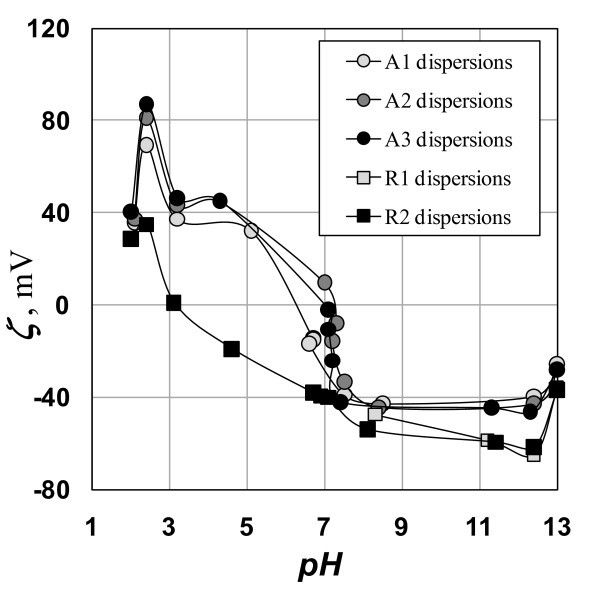
**Acidobasic adjusting of *ξ*-potential**. Individual nanopowders are specified in Table 1.

In preliminary experiments, 0.02 g of a nanopowder was added into 25 mL of each base solution. The flask with a suspension was treated for 30 min in a 40-kHz ultrasonic bath with a nominal acoustic power of 30 kW m^-3^. The samples were then tested using DLS technique (Zetasizer Nano ZS - Malvern Instruments) to determine the zeta potential, *ξ*. Actual values of pH, see Figure 1, slightly differ from idealized log-linear estimates (dotted line in Figure 1) even for a series of the base solutions. This difference is caused by dissociation of water and hydrated TiO_2_, as well as by the presence of dissolved CO_2 _(around *c*_NaOH _= 10^-5 ^mol/L). The resulting ξ-potentials dependent on the actual measured pH values are plotted in Figure 2.

Assuming that the maximum stability of a TiO_2_-water dispersion, i.e., the highest resistance against sedimentation, can be achieved at the extreme values of *ξ*-potential [1], further ten samples (A1±, A2±, A3±, R1±, and R2±), were prepared to examine their particle size distribution using again the DLS technique; see also Table 2. The preparation of these samples differs from the preliminary procedure only in the utilization of a larger primary amount of nanopowder (2.5 g in 100 g of dispersion) and a longer ultrasonication time (24 h). An external cooling system was employed to keep the sample at a constant temperature of 23°C during ultrasonic treatment. After keeping the sample aside for next 8 h, the sediment (ranging from 5 to 90% of the original content of nanopowder) was withdrawn and weighed to determine the final real particle concentration, shown in Table 2.

**Table 2:** Parameters of the stable nanofluids

Sample	Powder	Base solution	pH	ξ (mV)	Conc. TiO_2 _(wt.%)	Conc. TiO_2 _(vol.%)
A1+	A1	10^-2 ^M HCl	2.4	69.4	1.2	0.31
A1-^a^	A1	10^-3 ^M NaOH	12.4	-42.7	0.8^b^	0.2^a^
A2+	A2	10^-2 ^M HCl	2.4	81.2	2.4	0.65
A2-^a^	A2	10^-3 ^M NaOH	12.4	-44.3	1.2*	0.3*
A3+	A3	10^-2 ^M HCl	2.4	87	1.4	0.36
A3-^a^	A3	10^-3 ^M NaOH	12.3	-44.5	0.8*	0.2*
R1+^a^	R1	10^-2 ^M HCl	2.4	-	0.2*	0.05*
R1-	R1	10^-2 ^M NaOH	12.4	-65.2	0.2	0.05
R2+^a^	R2	10^-2 ^M HCl	2.4	34.9	0.2^a^	0.05^a^
R2-	R2	10^-2 ^M NaOH	12.4	-61.6	0.3	0.07

Volumetric concentrations were calculated using the densities from Table 1.

^a^Unstable samples with rough estimates of particle concentration.

**Figure 3:**
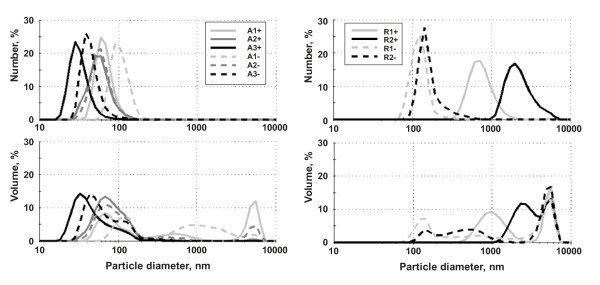
**Particle size distributions via DLS method**. Color and style of the curves identifies the samples, specified in Table 2. Note a large volumetric content of coarse particles in the anatase sample A1+ and in all the rutile samples. This is apparent in the volume-weighed distributions, while almost hidden in the number-weighed distributions.

The resulting particle size distributions, Figure 3, show remarkable differences in the behaviors of anatase- and rutile-based dispersions. While the anatase dispersions display the maximum content of the finest particles in acid media (A1+, A2+, and A3+), the rutile dispersions in acid media (R1+ and R2+) are much more coarse. In alkaline media, on the contrary, the anatase dispersions (A1-, A2-, and A3-) display a remarkable shift toward coarse clusters, whereas the rutile dispersions (R1- and R2-) become finer. As a matter of fact, the coarser dispersions (A1-, A2-, A3-, R1+, and R2+) settle rather fast, while the finer dispersions (A1+, A2+, A3+, R1-, and R2-) are stable for a few days. Only the stable dispersions were further subjected to rheological examinations using the AWS rotational viscometry.

### AWS rotational viscometry

**Figure 4:**
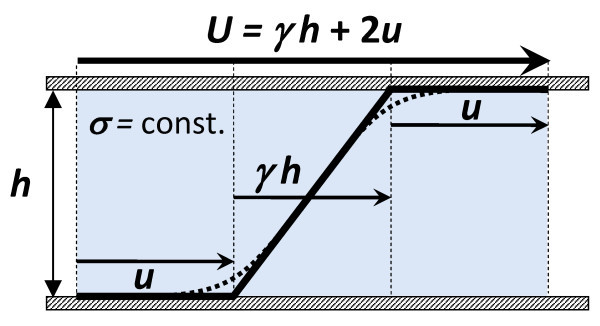
**Scheme of a shear flow with the AWS effect**. Dotted line - actual non-linear velocity profile observed at the constant shear stress *σ *due to the effect of a depletion layer of dispersion at the wall; Broken solid line - approximation of the actual velocity profile, introduced by the concept of AWS [18]. *U *= γ*h *+ 2*u *- macroscopic sliding velocity, m s^-1^; *h *- gap thickness, m; *u *- AWS velocity, m s^-1^; *γ *- bulk shear rate, s^-1^.

The concept of AWS effect from the viscometric viewpoint [17-19] is illustrated in Figure 4 for the simple shear flow between two mutually sliding parallel plates. A possible near-wall flow anomaly, resulting in a non-linear velocity profile under constant shear stress σ, is represented by the apparent slip velocity *u*. The only experimentally available kinematic quantity, the sliding velocity *U*, determines the apparent shear rate γ_app _≡ *U*/*h *(or γ_app _= *ΩR*/*h *for the Couette flow in a narrow gap *h *between two coaxial cylinders), which is expressed as a sum of the bulk flow and wall slip contributions, as follows:(1)

Two material functions, the bulk fluidity *φ*[*σ*] ≡ *γ*/*σ *and the Navier slip coefficient *χ*[*σ*] ≡ *u*/*σ*, are constant in many cases [17-19]. The flow and slip effects can be distinguished through a series of viscometric experiments, in which the gap thickness *h *is systematically varied whereas the shear stress *σ *is kept constant. This is the essence of AWS viscometry.

### Rotational viscometer with a KK sensor

**Figure 5:**
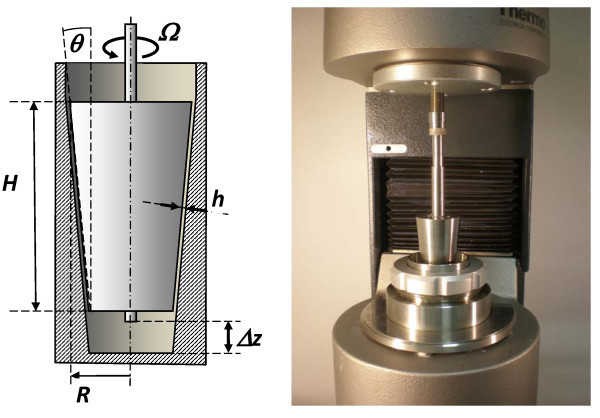
**KK sensor for AWS viscometry operating under HAAKE RS 600 rotational viscometer**. Common geometry parameters for all the KK sensors: *H *= 60 mm, *R *= 17.5 mm, cot(θ) = 10. The actual gap thickness *h *is adjustable through axial shift Δ*z*, see Equation 2. When applying Equation 1 for description of the AWS effect, take Ω*R *= *U*.

The experimental realization of AWS viscometry needs a series of sensors of different and well-calibrated hydraulic radii (tube radius in the capillary viscometry, gap thickness between cup and bob in the rotational viscometry, etc.). The novel KK-type sensor for the rotational AWS viscometry [19], shown in Figure 5 complies with this need by means of an axial shift facility for adjusting Δ*z *and, subsequently, the gap thickness *h *is given by(2)

where *h*_0 _corresponds to *h *at the starting position Δ*z *= 0. Both the working surfaces of the sensor are the coaxial cones of the same cone angle θ, as in a Morse clutch. The gap thickness can be adjusted over a broad range of 100-2500 μm with substantially (ten times) higher accuracy than for the plate-plate (PP) sensor. At the same time, the KK sensor displays much lesser edge effects and better reproducibility. In many applications, it is important to note that the measurements with a varied gap thickness can be made without refilling samples.

The fully automated rotational rheometer HAAKE RS 600 has been used both for driving the KK sensors and for data acquisition. When operating the KK sensor under HAAKE software *RheoWin*, it is appropriate to identify it with a PP-type sensor. Primary data in the text files, generated by the HAAKE *RheoWin *software, were further treated using a home-made software *AWSWork*, described in [19].

### Correction on centrifugal effects in AWS rotational viscometry

The original theory [19] of the KK sensors ignores possible inertia effects at the edges of rotating spindle. An additional correction *E *of the shear stress on inertia was until now considered only for the standard cylinder-cylinder Z40 DIN sensor [20]. This result can be rearranged to a local edge correction for a single semi-infinite cylinder by radius *R *rotating with a speed Ω in an infinite coaxial cylindrical vessel by radius *R *+ *h = R*(1 + κ), filled with a Newtonian liquid of kinematic viscosity υ = 1/(φρ):(3)

where κ ≡ *h*/*R*, *Re *≡ Ω*R*^2^*/υ*, *a *= 7.0 × 10^-4^, and *b *= 2.7 × 10^-4^.

For a KK-type conical spindle, the local edge effects are related to different radii at the both fronts, *R *and λ*R*, respectively, with a common *h *and λ = 1 - tan(*θ*) *H*/*R*, Figure 5. The final correction on centrifugal effects can be approximated for Newtonian liquids by the formula:(4)

## Results and discussion

### Stability and texture of dilute nanofluids

All the TiO_2 _dispersions, prepared in the described way, were partially settling down. The concentrations of the final stable dispersions depend on the base solution used, individual nanopowder, and dispersion procedure.

**Figure 6:**
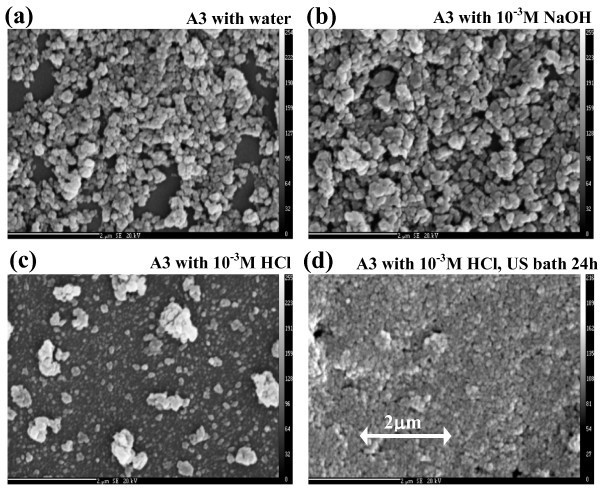
**Examples of SEM images of dried samples**. The representative photographs were selected for each tested sample. In contrast to the samples **(a, b)**, the samples **(c, d) **contain a major part of the nanopowder in the form of fine particles. In addition, the long-time ultrasonification, see sample **(d)**, breaks-up the remaining clusters apparent in sample **(c)**. The specification of A3 nanopowder is given in Table 1.

The series of images in Figure 6 illustrates the influence of the dispersion procedure and base solution on the texture of several dispersions of the nanopowder A3. The photographs were obtained using the SEM imaging technique (Cameca SX100), applied to the samples of the dried drops. In conclusion, the particles of the nanopowder A3 were better dispersed in the acidic solution than in the neutral or alkaline one (compare Figure 6a, b, c). The clusters remaining in the acid dispersion were broken up during the ultrasonic treatment (compare Figure 6c, d).

The influence of pH on the quality of dispersions was observed for all the tested dispersions via DLS technique. It can be seen from the number and volume-weighted particle size distributions (Figure 3) that anatase nanopowders disperse better in the acid solutions while rutile ones in the alkali solutions. The finer the dispersion the higher the concentration in the final stable samples.

### AWS rotational viscometry

Rheological measurements were conducted using the AWS rotational viscometry on the HAAKE RS 600 commercial instrument with a series of home-made KK sensors. Basic characterization of the examined samples is given in Table 2. As the AWS effect can depend on the material type and roughness of confining surfaces of the sensors, four different KK sensors were used, see Table 3. For the each combination sample - KK sensor, a series of individual viscometric measurements was made, covering the range of shear stress σ ∈ 0.05-5 Pa and the range of gap thickness *h *∈ 150-500 μm. In the final data treatment, including the inertia correction according to Equations 3 and 4, the primary data with σ > 1 Pa or *h *> 300 μm were disregarded (errors due to inertia effects over 5%). Uncorrected AWS data on φ and χ, not shown here, display remarkable dependence on σ, evoking a shear-thickening behavior. However, the correction of primary data on inertia effects shows that this dependence is only an experimental artifact.

**Table 3:** The KK sensors for AWS rotational viscometry

Sensor	Material	*h*_0 _(μm)
KK01	Stainless steel	173.5 ± 2
KK02	Titanium	134.5 ± 2
KK03	Eloxed dural	131.5 ± 2
KK04	Sand-blasted stainless steel	150.6 ± 2

All sensors share the nominal dimensions *R *= 17.5 mm; *H *= 60 mm; cot *θ *= 10.

The minimum gap thicknesses *h*_0 _were determined by calibrations with water.

The AWS data were further treated to separate the flow and slip contributions and to identify the corresponding material functions *φ*[*σ*] and *χ*[*σ*]. The resulting fluidities, given in Table 4, do not deviate from that of pure water by more than 3%. Statistical estimates of the slip extrapolation length *β *[19],(5)

**Table 4:** Results of rheological measurements at 23°C

Sample	Fluidity *φ *(Pa^-1 ^s^-1^)									
	
	KK01		KK02		KK03		KK04		All sensors	
	
	Avg	Dev	Avg	Dev	Avg	Dev	Avg	Dev	Avg	Dev
A1+	1032	16	1026	47	1049	10	1031	11	1036	10
A2+	1045	6	1041	6	1045	6	1041	10	1043	2
A3+	1001	10	1028	8	1022	18	1009	40	1015	12
R1-	1018	19	1031	22	1060	13	1069	14	1045	24
R2-	1042	10	1045	31	1073	12	1030	45	1048	18
Water	1033	17								

Avg & Dev, average and standard deviation for a given data series.

given in Table 5, indicate the mean values about zero with uncertainty about ±2 μm. This is in a good agreement with the estimate of instrumental uncertainty Δ*h *of the adjustable gap thickness *h*, given in Table 3. Possible slip effects in all the studied samples are therefore quite negligible in comparison with the instrumental uncertainty.

**Table 5:** Results of rheological measurements at 23°C

Sample	Slip extrapolation length *β *= *χ*/*φ *(μm)							
	
	KK01		KK02		KK03		KK04	
	
	*Avg*	*Dev*	*Avg*	*Dev*	*Avg*	*Dev*	*Avg*	*Dev*
A1+	2	2	0	2	6	2	0	2
A2+	2	2	0	2	2	2	2	2
A3+	9	5	1	2	3	3	5	5
R1-	3	3	5	3	5	5	5	5
R2-	6	4	9	2	7	3	4	4
Water	0	2						

Avg & Dev, average and standard deviation for a given data series.

**Figure 7:**
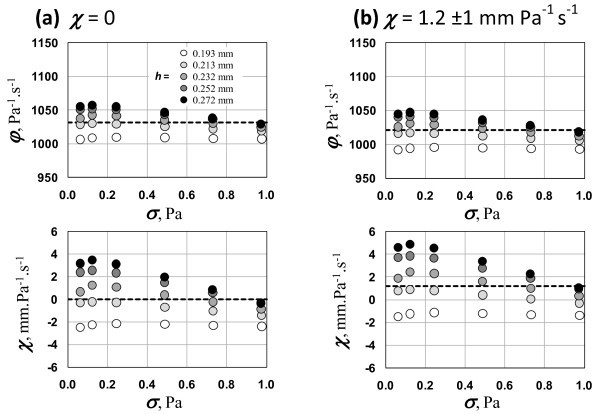
**Example of treating primary AWS data**. The example corresponds to sample A1 in KK01 sensor: **(a) **using constitutive model with no AWS (zero slip coefficient); **(b) **using constitutive model with adjustable constant slip coefficient.

The absence of slip effect is illustrated also in Figure 7, where the AWS data are fitted on two different constitutive models according to Equation 1, for details on the parametric filtration see [19]. Figure 7a shows the results obtained for the model with no-slip assumption, *χ *= 0, while the Figure 7b shows those for the model with adjustable but constant *χ*. Comparing of the both approaches shows that they provide nearly same estimates of the fluidity.

## Conclusions

AWS rotational viscometry with KK-type sensors represents a novel technique suitable for testing microdisperse fluids in the presence of slip effects.

Several dilute TiO_2_-water stable nanofluids with an optimized pH (via *ξ*-potential) are used to demonstrate the capability of this instrumentation to detect possible slip effects even in low-viscosity liquid samples. The tested stable colloidal samples differ in the nominal volumetric concentrations of nanoparticles, ranging from 0.07 to 0.7 vol.%.

The sensitivity of the AWS viscometric instrument on slip effects depends on the minimum available gap thickness and the accuracy of its adjustment. Within the given instrumentational limits, no slip effect was detected for the nanofluid samples examined for this investigation.

## Abbreviations

AWS: apparent wall slip.

## Competing interests

The authors declare that they have no competing interests.

## Authors' contributions

VP carried out experiments, and evaluations, including development of special software. JT participated as a consultant both in rheology and nanotechnologies. OW developed KK-sensors and the related theory. All the authors read and approved the final manuscript.
